# PW02-022 - Recurrent fever syndromes: multiple gene mutations

**DOI:** 10.1186/1546-0096-11-S1-A162

**Published:** 2013-11-08

**Authors:** M Bohm, P Dolezalova, H Lachmann, C Modesto, I Touitou, P Woo, M Finetti, V Hentgen, L Cantarini, F De Benedetti, R Gallizzi, L Obici, R Manna, E Gallo, N Ruperto, M Gattorno

**Affiliations:** 1 Charles University in Prague, Prague, Czech Republic; 2 University College Medical School, London, UK; 3 Hospital Valle de Hebron, Barcelona, Spain; 4 Hôpital Arnaud de Villeneuve, Montpellier, France; 5 Great Ormond Street Hospital, London, UK; 6 Istituto Giannina Gaslini, Genova, Italy; 7 Centre de reference national des maladies auto-inflammatoires, Paris, France; 8 University of Siena, Siena, Italy; 9 Ospedale Pediatrico Bambin Gesù, Roma, Italy; 10 Università di Messina, Messina, Italy; 11 Fondazione IRCCS Policlinico S. Matteo, Pavia, Italy; 12 Policlinico Gemelli, Roma, Italy; 13 Clinica Pediatrica Università di Torino, Torino, Italy

## Introduction

In patients with monogenic autoinflammatory diseases, the majority of detected mutations were found just in a single gene, either in homozygous, heterozygous or compound heterozygous state. However, in some patients mutations in several different genes have been detected. Clinical significance of this finding has yet to be established.

## Objectives

To describe clinical characteristics of patients with rare combinations of genetic mutations.

## Methods

Seventy-seven centres from 33 countries have been contributing to an international secured web-based registry for autoinflammatory diseases (EUROFEVER), hosted by the PRINTO website (Paediatric Rheumatology International Trial Organisation, http://www.printo.it).

The registry collects anonymised demographic, clinical, laboratory and molecular genetic data on patients with autoinflammatory diseases. Complete clinical information on 1868 consecutive children was available.

## Results

In 31 patients (1.7%), the combination of mutations in two different genes, and in one patient in three genes was found with following distribution of clinical diagnoses: Cryopyrin-Associated Periodic Syndromes (CAPS)=9, Tumor necrosis factor (TNF)-Receptor Associated Periodic Syndrome (TRAPS)=7, Familial Mediterranean Fever (FMF)=5, mild Mevalonate Kinase Deficiency (MKD, also known as Hyper IgD Syndrome (HIDS))=2, undefined=9. Out of these patients 18 (56%) carried one high penetrance mutation each. The prevalence of high penetrance mutations among clinically defined groups was most prominent within the CAPS phenotype (8/9) followed by FMF (3/5), MKD (1/2) and TRAPS (3/7). In patients with low penetrance mutations in one of the 4 relevant genes the second mutation was found in one of the remaining 3 genes with the following characteristics: low penetrance (5/14), polymorphisms (3/14), unknown (4/14). In remaining 2 patients the combination of unknown penetrance mutation in one gene and polymorphism in the other was found (both with undefined phenotype).

## Conclusion

As the availability of molecular genetic analysis for patients with recurrent fever syndromes increases, the amount of detected mutations in more than one gene will grow. The data currently available suggest that the high prevalence mutations overrule the clinical picture of the disease in majority of patients, though clinical significance of second mutations will have to be evaluated in larger patient series.

## Disclosure of interest

None declared.

